# Taste-Masked Diclofenac Sodium Microparticles Prepared by Polyelectrolyte Complexation: Formulation Using Different Fatty Acids and Taste Evaluation by Human Panel

**DOI:** 10.3390/pharmaceutics17111411

**Published:** 2025-10-30

**Authors:** Okhee Yoo, Sharmin Sultana, Britta S. von Ungern-Sternberg, Lee Yong Lim

**Affiliations:** 1 Department of Pharmacy, School of Health and Clinical Sciences, University of Western Australia, Perth, WA 6009, Australia; okhee.yoo@uwa.edu.au (O.Y.); sharmin.sultana@uwa.edu.au (S.S.); 2 Institute for Paediatric Perioperative Excellence, The University of Western Australia, Perth, WA 6009, Australia; britta.regli-vonungern@health.wa.gov.au; 3 Centre for Optimisation of Medicines, School of Health and Clinical Sciences, University of Western Australia, Perth, WA 6009, Australia; 4 Wesfarmers Centre for Vaccines and Infectious Diseases, The Kids Research Institute, Nedlands, WA 6009, Australia; 5 Perioperative Medicine Team, The Kids Research Institute Australia, Nedlands, WA 6009, Australia; 6 Division of Emergency Medicine, Anaesthesia and Pain Medicine, Medical School, The University of Western Australia, Perth, WA 6009, Australia; 7 Department of Anaesthesia and Pain Medicine, Perth Children’s Hospital, Nedlands, WA 6009, Australia

**Keywords:** diclofenac sodium, polyelectrolyte complex, taste masking, eudragit EPO, fatty acid, pediatric formulation, palmitic acid, lauric acid, stearic acid, myristic acid

## Abstract

**Background/Objectives**: Paediatric patients continue to lack access to age-appropriate oral medicines for their treatment and have to depend on the off-label use of medicines approved for adults, which compromises dosing accuracy and exposes children to unpleasant bitterness. Building on previous proof-of-concept work with flucloxacillin sodium, this study investigated the effects of fatty-acid chain length on the formation, stability, dissolution, and sensory acceptability of diclofenac sodium (DS)–Eudragit® EPO (EE)–fatty acid (FA) polyelectrolyte complexes (PECs). Four saturated fatty acids, lauric (C12), myristic (C14), palmitic (C16), and stearic acid (C18), were evaluated at stoichiometric equimolar DS:EE:FA ratio (1:1:1). **Methods**: PEC microparticles were prepared by solvent evaporation. A stability-indicating RP-HPLC assay was developed and validated according to ICH guidelines to quantify DS content. Drug content and stability were monitored over 3 months at ambient storage. In vitro dissolution was performed in pH 5.5 medium at 37 °C. Taste acceptability and willingness to take again was assessed with 25 healthy adult volunteers using 11-point scale. **Results**: All PECs retained >90% of expected drug content after 3 months. Compared with neat DS, PECs markedly suppressed early drug release (32–39% vs. 94% at 2 min) but achieved >87% cumulative drug release in 60 min. Sensory evaluation showed significant differences across samples (*p* < 0.001): neat DS was least acceptable (20.8% willing to take again), while DS-EE-PA was most acceptable (92%), followed by DS-EE-SA and DS-EE-MA. DS-EE-LA was least favoured among PECs. **Conclusions**: Fatty-acid chain length influenced PEC formation and taste acceptability, but not the PEC stability and drug dissolution profile. Palmitic acid (DS-EE-PA) offered the best overall profile and represents a promising candidate for further development of paediatric-appropriate diclofenac formulations.

## 1. Introduction

Paediatric patients continue to lack access to age-appropriate oral medicines for their treatment and have to depend on the off-label use of medicines approved for adults [1,2]. The manipulation of approved medicines for use in children may result in inaccurate dosing, unknown bioavailability, and exposure to aversive drug tastes that then contribute to poor therapeutic outcomes [3]. There is, therefore, an urgent need to formulate taste-masked oral formulations that are stable on storage, capable of providing accurate dosing, and safe to be swallowed by even very young children. 

We have previously provided proof of concept that combining flucloxacillin sodium (FS) with Eudragit® EPO (EE) and palmitic acid (PA) yields polyelectrolyte complex (PEC) microparticles that may provide taste masking for FS [4,5]. The PEC was obtained by dissolving the three ingredients in appropriate solvent systems, mixing the solutions to allow for complexation, and casting the resultant suspension onto drying trays to yield a powder of microparticles following complete solvent evaporation at ambient conditions. Complexation occurs via three-way spontaneous, entropy-driven acid-base reactions that involved the carboxylic acid of PA and the tertiary amine group of EE to form PA-COO^-^, which reacted with the Na^+^ counterion of FS to form sodium palmitate, while the protonated EE interacted with the ionised FS [6]. FS was chosen as a model drug because of its highly aversive taste and poor acceptance among paediatric patients. The choice of PA, a C16 saturated alkane, as fatty acid was informed by several considerations, including its availability at relatively low cost compared to newer polar lipids and its moderately low melting point (~63 °C), which facilitates processing at temperatures suitable for heat-sensitive drugs. EE is a cationic polymer that dissolves at a pH below 5, and it has been successfully applied as coating for taste-masked solid dosage forms [7,8]. Formulating FS with EE and PA provided chemically stable ternary microparticles that were effective at suppressing FS release in simulated oral cavity conditions (pH ~6–7) while allowing rapid FS release into simulated gastric fluid (pH < 5) [4,5,9]. Thus, the PEC microparticles may inhibit FS release for interaction with taste receptors in the oral cavity, while the dissolution of EE in the gastric fluid will allow for complete FS release for absorption in the lower gastrointestinal tract.

Taste-masking effectiveness of the FS-EE-PA microparticles had been evaluated using surrogate in vitro dissolution data [4,5,9] and not with human taste panels, the gold standard for evaluating the taste of oral medicines. Transferability of application of the PEC design principles has also not been tested, in that, besides FS, it has not been assessed using another drug in the sodium salt form. Additionally, there are several saturated fatty acids that may be more successfully applied for this platform than PA. Indeed, EE has been reported to form soluble ionic complexes with stearic acid, a C18 fatty acid [10]. Given that an ideal taste-masking drug delivery platform should be evaluated by human taste panels to confirm its taste-masking effectiveness for a range of drugs with known aversive tastes, there is, therefore, scope to evaluate the PEC platform for formulation with other drugs and fatty acids, as well as to confirm its taste-masking effectiveness using human taste panels. 

**Figure 1:**
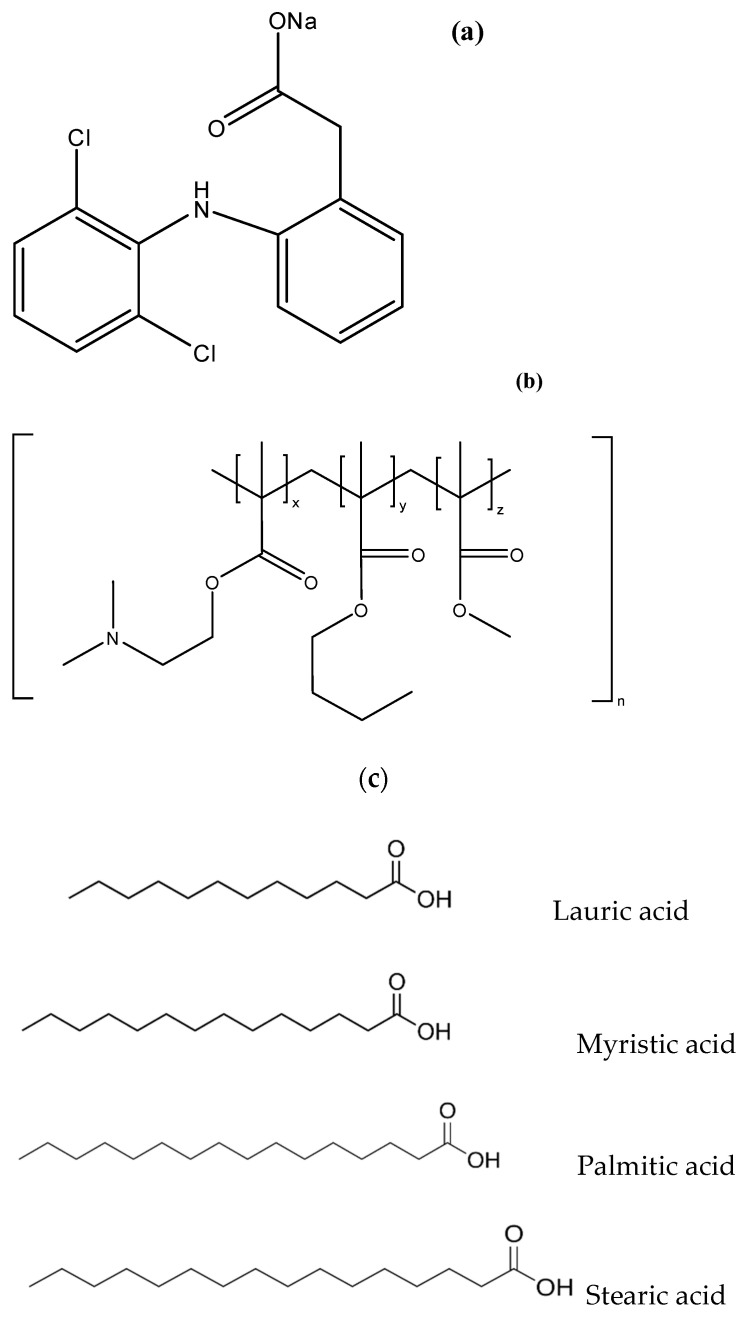
Chemical structures of (**a**) diclofenac sodium, (**b**) Eudragit EPO and (**c**) fatty acids used to formulate the polyelectrolyte complex microparticles.

The objective of this study was to apply the PEC design principles to diclofenac sodium (DS) (Figure 1), EE and saturated fatty acids of different chain lengths, and to evaluate the resultant PEC microparticles for taste-masking effectiveness and storage stability. DS is a bitter-tasting nonsteroidal anti-inflammatory drug prescribed for pain relief and frequently used off-label in children due to a lack of licenced oral paediatric formulations [11]. Apart from PA (C16), three other saturated fatty acids were also employed in this study, namely lauric acid (C12), myristic acid (C14), and stearic acid (C18) (Figure 1). The PEC microparticles formulated with each fatty acid was assessed for taste by a panel of young adult human volunteers to determine whether the fatty acids can equivalently interact with EE and DS to produce taste-masked microparticles. Our hypothesis was that ternary complexes of DS and EE with the different saturated fatty acids would achieve similarly stable and effective taste-masked microparticles suitable for use in paediatric patients.

## 2. Materials and Methods

### 2.1. Materials

DS (USP grade) was provided by Medisca (Sydney, NSW, Australia). Eudragit EPO was kindly gifted by Evonik Industries (Essen, Germany). Lauric acid and myristic acid were sourced from Sigma-Aldrich (Darmstadt, Germany), while palmitic acid was from Acros Organics (Geel, Belgium) and stearic acid was from PharmAust (Malaga, WA, Australia). All fatty acids had a purity greater than 98%. HPLC-grade methanol was obtained from Honeywell Burdick and Jackson (Morristown, AZ, USA). Potassium dihydrogen orthophosphate was procured from AnalaR (London, UK). Deionised water was employed for all experimental procedures and filtered under vacuum for use in HPLC analysis.

### 2.2. Methods

#### 2.2.1. Preparation of Polyelectrolyte Complexes

The DS PEC microparticles were prepared using stoichiometric equimolar ratio of DS:EE:fatty acid, the ratio having been established by optimisation studies of the FS-EE-PA PEC using the mixture design [4,5,9]. Method for preparing the DS PEC microparticles also employed the fabrication methodology previously validated for the FS-EE-PA PEC microparticles [4,5,9]. A total of four ternary complexes were prepared (Table 1). Each PEC was prepared using equimolar ratio of DS, EE, and one of the four saturated fatty acids: lauric acid (LA), myristic acid (MA), palmitic acid (PA), and stearic acid (SA). The weight ratio corresponding to molar ratio was calculated based on the 1:1:1 molar ratio of carboxylic acid of DS: tertiary amine of a functional monomer of EE: carboxylic acid of fatty acid. The molecular weight of EE was an approximation provided by the manufacturer, and the EE contained a nitrogen content equivalent to 4–6% of its dry mass [12]. The number of tertiary amine groups per gram of EE was estimated using the lower value of 4% (EE 350 g/mol) while the weight of fatty acid based on the matching number of functional group to tertiary amine groups per gram of EE was estimated using the upper value (6%, 233 g/mol) of the approximation [9]. Thus, the equimolar ratio was equivalent to the weight ratio of approximately (1.59:1.59:1) *w*/*w*/*w* for DS:EE:LA, (1.39:1.39:1) *w*/*w*/*w* for DS:EE:MA, (1.24:1.24:1) *w*/*w*/*w* for DS:EE:PA and (1.12:1.12:1) *w*/*w*/*w* for DS:EE:SA (Table 1).

To prepare a batch of PEC, 0.3181 g of DS was dissolved in 8 mL ethanol, while 0.3181 g of EE and the appropriate weight of specified fatty acid (Table 1) were co-dissolved in 5 mL ethanol under magnetic stirring. The DS solution was poured into the EE-fatty acid solution, and the mixture was stirred at 100 rpm for one hour. The resultant suspension was spread as a thin layer onto a ceramic plate and allowed to dry overnight at ambient conditions. The dried sample was collected, ground using a pestle and mortar, and sieved (aperture size 212 μm, Glenammer Laboratory Test Sieves, Ayr, Scotland, UK) to obtain particles of relatively uniform sizes. To ensure complete solvent evaporation, the powder was stored at ambient temperature in a 10 mL glass vial covered with a perforated parafilm, and its weight monitored daily until no further change in weight was observed (approximately 7 days). The parafilm was then replaced with plastic caps, and the samples were stored at room temperature, protected from light. All PEC formulations were prepared in triplicate batches for analysis.

#### 2.2.2. Storage Stability Assessment

The stability of the PEC formulations over a three-month period was assessed by analysing the DS content and DS dissolution profile at baseline, and at one month and three months of storing the PEC microparticles in capped glass vials under ambient room conditions (approximately 25 °C), protected from light. Baseline was defined as 7 days post-preparation, when the PEC microparticles after complete solvent evaporation were transferred to capped vials for storage.

#### 2.2.3. Diclofenac Sodium Content Analysis

DS was quantified by high-performance liquid chromatography (HPLC), using the method published in the British Pharmacopoeia, with modifications. The analysis was performed on an HPLC system (Ultimate 3000, Thermo Scientific, Perth, Australia) equipped with diode-array detector and ZORBAX Eclipse XDB-C18 column (5 μm, 150 mm × 4.6 mm, Waters Australia, Rydalmere, NSW, Australia). The mobile phase consisted of methanol and an aqueous buffer of 0.2% (*v*/*v*) phosphoric acid (80:20 *v*/*v*). Analysis was conducted at a flow rate of 1.0 mL/min, injection volume of 10 µL and run time of 10 min. DS was detected at a wavelength of 282 nm. Chromatographic data were processed using the Chromeleon Chromatography 7 software (version 7.2.8, Sunnyvale, CA, USA).

The HPLC assay was validated for linearity, specificity, precision, and accuracy according to the ICH guidelines [13]. Linearity was confirmed by correlation coefficient (r^2^) of 0.999 between peak area and concentration of DS. A 100 µg/mL DS stock solution in methanol was serially diluted with methanol to obtain calibration standards with a concentration range between 10 and 50 µg/mL. Specificity was assessed by dissolving a mixture of the four fatty acids (2 mg each) together with EE (2 mg) in methanol and diluting to 100 mL, prepared both with and without 2 mg of DS, followed by HPLC analysis to determine whether any excipient peaks interfered with the DS peak. Accuracy, expressed as percent drug recovery, was evaluated by triplicate analyses at three concentration levels (12.5, 25, and 47.5 µg/mL). Precision, expressed as relative standard deviation (%RSD), was determined at five concentration levels (10, 20, 30, 40, and 50 µg/mL), with each concentration analysed in triplicates within a day and over 3 days to determine the intra-day and inter-day precisions, respectively. To establish stability-indicating capability, 2 mL aliquots of a DS solution (500 µg/mL in methanol) were heated at 100 °C for 1 h with acid (2 mL of 1.0 M HCl) or base (2 mL of 1.0 M NaOH) or exposed to UV light for 1 h with 2 mL of 30 % *v*/*v* hydrogen peroxide. The treated samples were analysed after diluting with methanol to 100 µg/mL, with the acid and base samples also adjusted to neutral pH by adding 1.0 M NaOH or 1.0 M HCl, respectively. All samples for HPLC analysis were filtered (0.45 µm, Agilent, Lexington, MA, USA) and 1 mL aliquots were transferred into HPLC vials for analysis.

To quantify DS in the PEC, approximately 5 mg of sample (equivalent to 2 mg of DS) was accurately weighed into a 100 mL volumetric flask, dissolved in 50 mL of methanol with sonication for 30 min, then made up to volume with methanol. A 1 mL aliquot of the solution was filtered through a 0.45 µm nylon syringe filter and transferred to an HPLC vial for analysis. Calibration standards of DS were prepared in methanol at concentrations ranging from 10 to 50 µg/mL.

#### 2.2.4. In Vitro Drug Dissolution Profile

In vitro drug dissolution studies were performed using accurately weighed amounts of PEC sample equivalent to 5 mg of DS and 90 mL of dissolution medium comprising deionised water adjusted to pH 5.5 with acetic acid [14]. A pH of 5.5 was chosen to facilitate the dissolution of both EE and DS from the PEC particles. The dissolution experiments were conducted at 37 ± 0.5 °C and a stirring rate of 100 rpm. Aliquots of 1 mL were withdrawn from the dissolution medium at predetermined intervals (0, 2, 5, 15, 30, 45, and 60 min), filtered through 0.45 µm nylon filters, and analysed using the validated HPLC method. Each withdrawn aliquot was replaced with an equal volume of fresh dissolution medium. Cumulative drug release was calculated and plotted as a function of time to generate the dissolution profile for each PEC formulation.

#### 2.2.5. Taste Evaluation

Taste evaluation protocols were approved by the UWA Human Research Ethics Committee (2023/ET000880). A total of 25 healthy young adults aged 18 to 35 years were recruited. The exclusion criteria were a known allergy to diclofenac or any other study ingredients and a history of gastrointestinal diseases, pregnancy, breastfeeding, or smoking. All participants provided written informed consent prior to participation in the taste trial.

**Figure 2:**
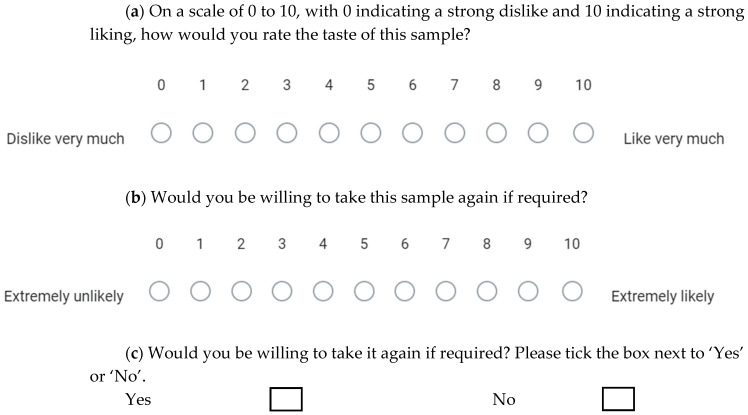
Methods applied to collect participant response in the taste assessment of the polyelectrolyte complex formulations. (**a**) Numeric 11-point scale to assess sample taste acceptability, which ranges from 0 = dislike very much to 10 = like very much. (**b**) Numeric 11-point scale to assess willingness to take the sample again, which ranges from 0 = extremely unlikely to 10 = extremely likely. (**c**) Binary response to assess willingness to take the sample again, recorded as a Yes/No response.

Each participant was asked to taste five samples: 10 mg pure DS (control) and the four types of PEC microparticles (25.5 mg DS-EE-LA, 26.3 mg DS-EE-MA, 27.2 mg DS-EE-PA, and 28.1 mg DS-EE-SA) each containing 10 mg of DS. The samples were presented neat to each participant using disposable wooden spoons and in a randomised order determined by a computer-generated block randomisation schedule. Using a “suck and spit” method, the participants were instructed to keep each sample in their mouth for 10 seconds before spitting it out to minimise drug intake. To neutralise any lingering taste, a 10-minute waiting period was enforced between tasting of samples, and participants were provided with mineral water and plain crackers to cleanse their palates. Immediately after tasting each sample, the participants were asked to rate their taste preference using an 11-point scale that ranged from “Dislike very much” (0) to “Like very much” (10) (Figure 2a). The participants also rated their willingness to take the medicine again using a separate 11-point scale that ranged from “Extremely unlikely” (0) to “Extremely likely” (10) (Figure 2b). Finally, the participants were asked to rate their willingness to take the medicine again using a simple binary Yes/No response (Figure 2c).

#### 2.2.6. Statistical Analysis

All statistical analyses were conducted in R (version 4.1.3, R Foundation for Statistical Computing, Vienna, Austria) [15]. Data are presented as mean ± standard deviation unless otherwise stated, and significance was established at *p* < 0.05. Residual drug content was compared across storage times (0, 1, and 3 months) for each formulation using one-way ANOVA, followed by Bonferroni-adjusted post hoc comparisons. Cumulative drug release profiles were reported as mean ± 95% confidence interval (CI, *n* = 3). Between-group differences in cumulative drug release at 60 min were assessed by one-way ANOVA with Tukey-adjusted pairwise comparisons. The time to 80% release (t_80_) was estimated by linear interpolation. The numeric taste scores and “willingness to take the sample again” scores, both measured on 0–10 scales, were analysed using the Friedman test and, when found significant, pairwise Wilcoxon signed-rank tests with Bonferroni adjustment were performed. The binary Yes/No responses for “willingness to take the sample again” were analysed using Cochran’s Q test, with significant results analysed further by McNemar pairwise comparisons and Bonferroni adjustment. Descriptive results were summarised as percentages, and 95% confidence intervals for proportions were calculated using the Wilson method.

## 3. Results and Discussion

**Figure 3:**
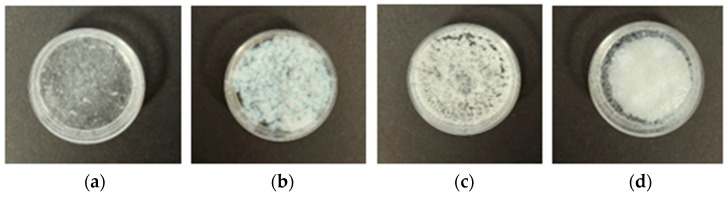
Appearance of diclofenac–Eudragit EPO–fatty acid (DS–EE–FA) complexes after 1 day of storage at room temperature post-production. Formulations were prepared with (**a**) lauric acid (DS-EE-LA), (**b**) myristic acid (DS-EE-MA), (**c**) palmitic acid (DS-EE-PA), and (**d**) stearic acid (DS-EE-SA).

PEC microparticles were successfully prepared with each of the four fatty acids using the method described in Section 2.2.1. However, markedly different results were observed when the complexation reaction was initiated by mixing the DS solution with the EE-fatty acid solution. With the shortest chain LA (C12) as fatty acid, the mixture remained translucent for at least 30 min of stirring before gradually turning turbid, and the opacity of the final suspension was much less intense compared to those obtained with the longer chain fatty acids (Figure 3a). By contrast, with the longest chain SA (C18) as fatty acid, pouring the DS solution into the EE-fatty acid solution led to instant phase separation, yielding a thick and homogeneous suspension with pronounced opacity and a cream-like texture (Figure 3d). With MA (C14) or PA (C16) as fatty acid, mixing the DS and EE-fatty acid solutions also yielded instant coagulation (Figure 3b,c), but the coagulates exhibited textures different to each other and to those seen with SA. The MA and PA suspensions were also not homogeneous, as larger sized coagulates were interspersed with fine particles on the liquid surfaces, and the turbidity of the suspensions were less intense compared with the SA suspension. These differences suggest that, while PEC formation was spontaneous for all the formulations, the fatty acid chain length influenced the kinetics of the complexation reactions. So long as sufficient time was allowed for completion of the complexation reactions, all four fatty acids ultimately yielded complexes that could be isolated as dry white powders and transformable into microparticles by grinding using a pestle and mortar. 

The mass change in the powders during storage was minimal after the first 24 h of drying. After this period, the samples were sufficiently dry to be ground and sieved through a 212 µm mesh. Daily monitoring showed that weight loss stabilised after Day 2, with total mass loss over the 7-day drying period remaining below 1%. From Day 3 onwards, no further measurable change was observed.

### 3.1. Stability Assessment: Diclofenac Sodium Content

**Figure 4:**
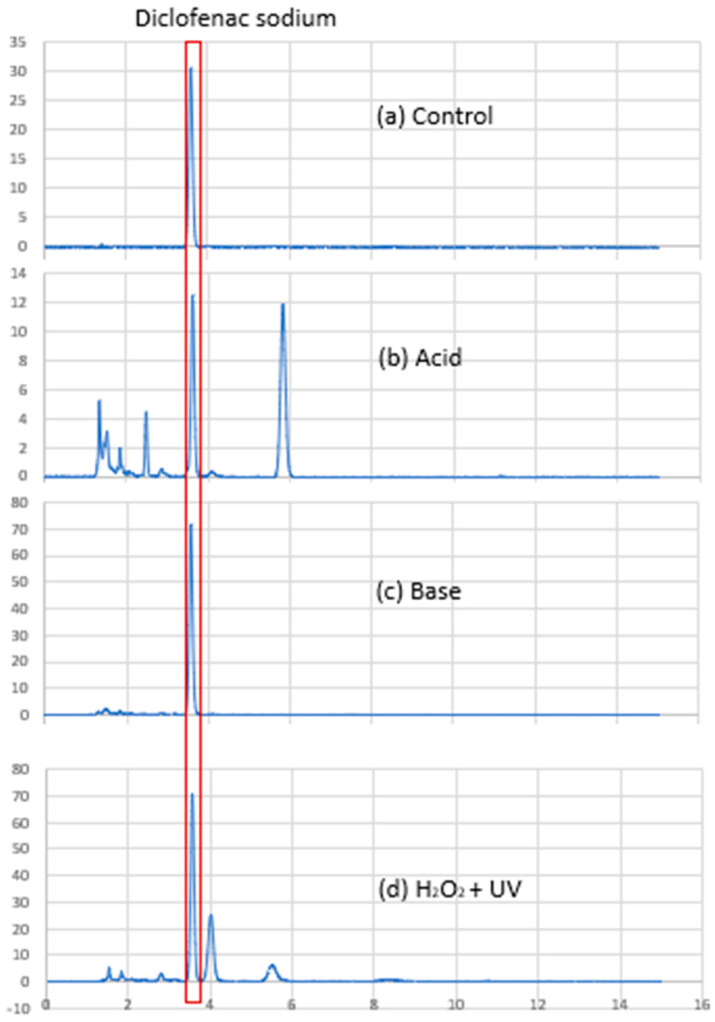
Chromatograms of diclofenac sodium (DS): (**a**) DS without treatment; (**b**) DS after 1 h heating at 100 °C in 1.0 M HCl; (**c**) DS after 1 h heating at 100 °C in 1.0 M NaOH; and (**d**) DS after 1 h exposure to UV light in 30% (*v*/*v)* hydrogen peroxide.

Using the HPLC method described in Section 2.2.1, DS was detected as a sharp and symmetrical peak with retention time at approximately 3.6 min (Figure 4a). Degradation peaks were observed in samples exposed to acid and H_2_O_2_, suggesting that DS was degraded by heating with acid and oxidation. The DS peak in these samples was well resolved from the degradation peaks (Figure 4), demonstrating that the assay was stability-indicating. The assay also demonstrated good linearity (R^2^ 0.9994) over the concentration range tested. Excipient peaks were detected at 4.2, 6.0, and 9.3 min but did not interfere with the DS peak at 3.6 min, confirming the specificity of the assay. The accuracy and precision values (Table 2) were well within the 15% acceptance limits specified by the ICH guidelines, indicating suitability of the assay for use to analyse the drug content of the PEC formulations at baseline and after storage [13].

HPLC analysis of DS content in the PEC microparticles indicated that the four PEC formulations contained ±10% of the labelled drug content at baseline; the range was 95.3% to 103.9% (Table 3). Upon storage at ambient temperature, the DS-EE-LA and DS-EE-PA samples showed significant changes in DS content in the first month of storage (*p* < 0.01) but not thereafter, with the DS content in DS-EE-LA increasing with storage while that in the DS-EE-PA decreased. Drug content in the DS-EE-SA sample also declined progressively across all time points (*p* < 0.01) while the DS-EE-MA showed no significant change in DS content over the 3 months of storage (*p* > 0.1). These changes in DS content could not be simply attributed to DS degradation in the PEC samples on storage because the DS content in the DS-EE-LA increased from 95.3 to 99.0%. Moreover, the range of DS contents in the four PEC samples stored under ambient temperatures was comparable to the range determined in the baseline PEC samples, with samples stored for 1 month showing 95.7 to 102.0% of labelled drug content, and samples stored for 3 months showing 95.9 to 102.7% of expected drug content. Thus, the different mean drug contents seen for the DS-EE-LA, DS-EE-PA, and DS-EE-SA samples might well be attributed to heterogeneity in drug loading in the microparticles during manufacture leading to significant differences in DS content for different samples drawn from the same batch of PEC microparticles. The differences notwithstanding, all four PEC formulations were found to retain >90% of labelled drug content, indicating satisfactory chemical stability of DS to the PEC manufacture process and subsequent storage at ambient temperature, protected from light.

### 3.2. Stability Assessment: In Vitro DS Dissolution Profile

**Figure 5:**
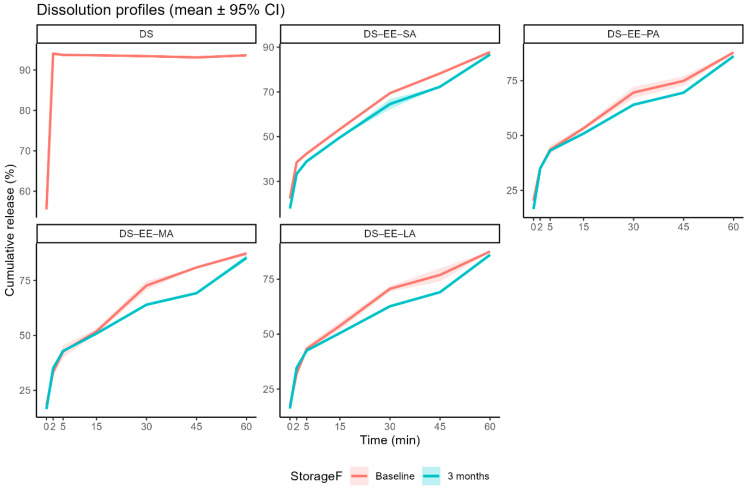
In vitro dissolution profiles of diclofenac sodium (DS) and DS–Eudragit EPO–fatty acid (DS–EE–FA) complexes prepared with stearic acid (SA), palmitic acid (PA), myristic acid (MA), and lauric acid (LA) at baseline (7 days post-preparation) and after 3 months of storage under ambient conditions. Dissolution was performed in 90 mL of deionised water adjusted to pH 5.5 with acetic acid at 37 °C under magnetic stirring (100 rpm) for 60 min. Data are presented as mean cumulative release (%) ±95% confidence interval (*n* = 3). Shaded ribbons represent the 95% confidence intervals around the mean profiles. DS was assessed at baseline only.

The control DS sample showed rapid dissolution rate, reaching almost complete dissolution (94.1 ± 0.5 %) in 2 min. Conversely, the PEC samples exhibited delayed release of DS, the percent DS load released from the four PEC formulations in the first 2 min ranging from 32.1% (DS-EE-LA) to 38.6% (DS-EE-SA). This suggests that complexation with EE and a fatty acid has the potential to reduce DS release during the short residence time in the oral cavity when the PEC microparticles are consumed orally. When the dissolution time was extended to 60 min for the four PEC formulations, no solid remnants were observed in the dissolution medium, suggesting complete dissolution of the PEC microparticles. HPLC analyses indicated that more than 87% of the DS loads in the four PEC microparticles were released in 60 min (Figure 5) and there was no significant difference in cumulative percent drug release between the different PEC formulations, and between the PEC formulations and the DS control (*p* > 0.05). This suggest that DS dissolution in the lower gastrointestinal tract, and by extension its bioavailability, is not likely to be affected by DS complexation with EE and the fatty acids. Moreover, with the dissolution medium having a pH of 5.5 whereas EE is reported to be soluble under acidic conditions of pH 5 or lower [12], the complete dissolution of the PEC microparticles in 60 min suggests that complexation with DS and fatty acids might enhance the aqueous solubility of EE at higher pH. 

Storage for 3 months at ambient conditions retarded the release of DS from the four PEC microparticles. The mean time for 80% of the drug load to be released (t_80_) ranged from 43.3 min (DS-EE-MA) to 50.8 min (DS-EE-PA) for baseline samples. While the t_80_ was not significantly different in values for the DS-EE-PA and DS-EE-LA (*p* > 0.05) microparticles after 3 months of storage at ambient temperature, the t_80_ values for DS-EE-SA (*p* = 0.00032) and DS-EE-MA (*p* = 0.00023) were significantly prolonged. However, the cumulative release of DS at 60 min remained high for all stored PEC samples, and they were comparable to the cumulative DS released at 60 min for the respective baseline samples.

### 3.3. Taste Evaluation

Using the cumulative amounts of DS released at 2 min and assuming a saliva volume of 1 mL prior to swallowing [16], it may be estimated that the four PEC microparticle formulations could produce DS concentration in saliva of ~2 mg/mL. A previous study involving 135 healthy young adults (mean age 23.8 ± 6.1 years) reported taste thresholds for DS of 1.008 ± 0.220 mM (0.32 ± 0.07 mg/mL) and 1.045 ± 0.240 mM (0.33 ± 0.08 mg/mL) using liquid-drop and paper-disc methods, respectively [17]. Another study had reported a lower DS taste threshold of 0.12 mg/mL [18]. Thus, the estimated salivary DS concentrations for all PECs at 2 min were above the reported taste detection thresholds for DS. Nonetheless, given the challenge of directly correlating in vitro dissolution data with human taste perception, a taste evaluation study was conducted using human taste panel members to assess the sensory characteristics of the four PEC formulations.

**Figure 6:**
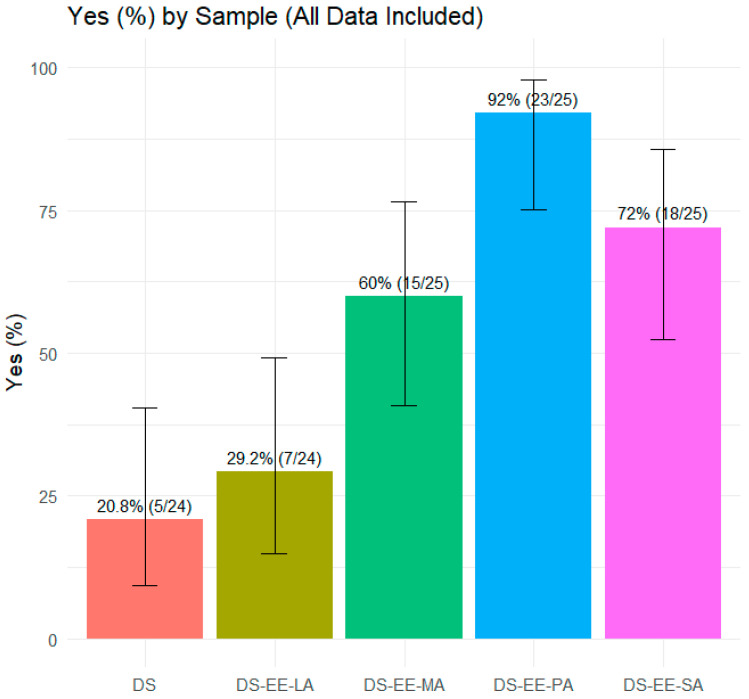
Proportion of participants willing to take each sample again (Yes/No). Data are presented as percentage of ‘Yes’ responses with Wilson 95% confidence intervals (*n* = 25). Samples tested were diclofenac sodium (DS) and DS–Eudragit EPO–fatty acid (DS–EE–FA) complexes with lauric acid (DS–EE–LA), myristic acid (DS–EE–MA), palmitic acid (DS–EE–PA), and stearic acid (DS–EE–SA).

**Figure 7:**
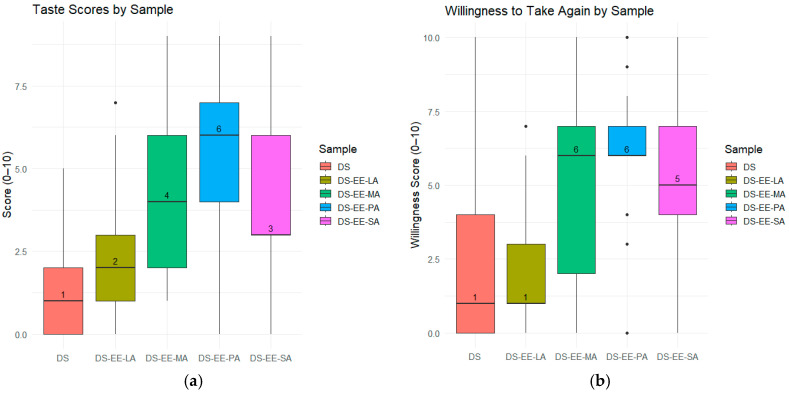
Participant evaluations of taste and willingness to take again across formulations. (**a**) Taste scores (0–10 scale; 0 = dislike very much, 10 = like very much); (**b**) Willingness-to-take-again scores (0–10 scale; 0 = extremely unlikely, 10 = extremely likely). Samples include diclofenac sodium (DS) and DS–Eudragit EPO–fatty acid (DS–EE–FA) complexes prepared with lauric acid (DS–EE–LA), myristic acid (DS–EE–MA), palmitic acid (DS–EE–PA), and stearic acid (DS–EE–SA). The horizontal line in each box represents the median score, which is also displayed as a numeric label, the box itself indicates the interquartile range (IQR, 25th to 75th percentiles), and the whiskers extend to show the rest of the distribution. Scores were provided by 25 participants for each sample.

A total of 25 healthy adult participants provided numerical ‘taste’ and ‘willingness to take again’ scores for the four PEC formulations and control DS sample. The ‘willingness to take again’ scores were used as a proxy for the overall acceptability of the formulations. Analysis by Friedman test demonstrated significant differences across the five samples for both ‘taste’ scores (χ^2^(4) = 54.3, *p* < 0.001) and ‘willingness to take again’ scores (χ^2^(4) = 57.8, *p* < 0.001). The control DS sample received the lowest taste scores and only 20.8% of the participants indicated that they would take it again if required (Figure 6). In contrast, DS-EE-PA received the highest taste scores, with an overwhelming 92% of participants reporting they would be willing to take it again (Figure 6 and Figure 7). Among the four PEC formulations, DS-EE-LA received the lowest ‘taste’ as well as ‘willingness to take again’ scores. Post hoc Wilcoxon signed-rank tests with Bonferroni correction showed that DS-EE-PA, DS-EE-SA, and DS-EE-MA were rated significantly higher than both the DS control and DS-EE-LA samples for ‘taste’ and ‘willingness to take again’ scores (*p* < 0.05). DS-EE-PA, DS-EE-SA, and DS-EE-MA did not differ significantly in taste scores. For ‘willingness to take again’ scores, DS-EE-PA was rated significantly higher than DS-EE-SA, whereas no significant differences were observed between DS-EE-SA and DS-EE-MA or between DS-EE-PA and DS-EE-MA.

DS-EE-LA was poorly accepted by the participants, and its ‘taste’ and ‘willingness to take again’ scores were closest to the control DS. The poor acceptability of DS-EE-LA might relate to the intrinsic sensory properties of lauric acid rather than the bitterness of DS, as all four PEC microparticles showed comparable DS release at 2 min in the dissolution study. The majority of participants described DS-EE-LA as having a coconut-like flavour, which is consistent with lauric acid being the predominant fatty acid in coconut oil [19]. Compared to longer chain fatty acids, LA is known to produce a stronger taste intensity with lingering burning, irritating, or spicy sensations [20]. By contrast, PA is regarded as having a less intense taste compared to fatty acids with C2 to C18 chain lengths [20], which might explain the significantly higher ‘taste’ and ‘willingness to take again’ scores for DS-EE-PA in the study. While statistically significant differences were detected among formulations, the results should be interpreted as indicative due to the modest sample size, and confirmation would require testing in a larger cohort with sample size determined statistically.

The ‘taste’ and ‘willingness to take again’ scores for the five samples were converted to Medicines Acceptability Score (MAS) using our previously published method [21]. Participants who scored in the range of 5–7 were categorised as passives, 8–10 were categorised as promoters and 0–4 were categorised as detractors. The percentage of participants categorised as passives, promoters, and detractors are tabulated in Table 4 based on the ‘taste’ and ‘willingness to take again’ scores they had provided for the five samples. MAS was the difference in percentages of participants categorised as promoters and detractors. While there were clear differences in the MAS values, the MAS for the four PEC formulations as well as the DS control samples were all negative and correlated to the percent ‘No’ response. It should be noted that the participants had evaluated the DS and PEC samples neat. While the DS-EE-PA had received higher ‘taste’ scores than DS and the PEC microparticles formulated with LA, MA and SA, most of the participants had still provided taste scores in the relatively neutral range (5–7) for DS-EE-PA. This suggests that, in their current microparticle form, the DS-EE-PA formulation had provided an acceptable but not necessarily pleasant taste perception, that is, it would be tolerable, as reflected by its high percentage of ‘Yes’ response to the ‘willingness to take again’ question. Acceptability of this formulation could likely be improved by transforming the microparticles into a final dosage form, such as an orodispersible tablet or chewable tablet, with appropriate excipients, such as sweeteners and flavouring agents.

Our previous work with FS, EE, and PA demonstrated the feasibility of polyelectrolyte complexation for taste masking. The present study extended this design concept to DS, another drug in sodium salt form and further explored the influence of fatty acid with different chain length on the effectiveness of complex formation and taste masking. While drug stability and overall dissolution behaviour remained broadly similar across the PEC formulated with LA, MA, PA, and SA, the chain length of the fatty acid clearly affected the kinetics of PEC formation and, more importantly, taste acceptability by a human taste panel. Among the four PEC microparticles, DS-EE-PA formulated with PA (C16) provided the most favourable qualities of storage stability, drug dissolution profile, and taste sensory profile. DS-EE-PA therefore represents the most promising candidate for further development into age-appropriate taste-masked medicinal products of DS for young children. Notably, although a prior study has evaluated EE for taste masking DS [18], the incorporation of a fatty acid to form taste-masked ternary microparticle complexes has not been previously reported. This study offers a novel opportunity to apply a simple method to develop a taste-masked DS microparticle formulation using generally regarded as safe (GRAS) excipients.

Further applicability of the polyelectrolyte complexation platform could be evaluated using other drugs in the salt forms, including diclofenac potassium, to assess whether the nature of the cation also influences complex formation and acceptability. In addition, the microparticles described here should be advanced into final dosage forms to enable flexible and safe dosing, ultimately supporting the development of paediatric-appropriate oral formulations.

## 4. Conclusions

This study demonstrated that PECs can be spontaneously formed between DS, Eudragit^®^ EPO, and saturated fatty acids of varying chain length. A stability-indicating RP-HPLC assay (ICH-compliant) was successfully developed and applied throughout. All formulations met chemical content targets at baseline and retained >90% of drug content after 3 months under ambient conditions.

Compared with neat DS, all PECs showed delayed drug release (t_80_) yet achieved >87% cumulative release within 60 min at baseline. Human evaluation showed that three of the four PECs achieved good taste acceptability, with the inherent taste of the fatty acid strongly influencing sensory outcomes. Among the tested lipids, palmitic acid (DS-EE-PA) provided the most acceptable quality characteristics, making it the most promising candidate for further development into paediatric-appropriate diclofenac formulations.

## Figures and Tables

**Table 1:** Labels and ingredient compositions for the four ternary polyelectrolyte complex formulations prepared in the study. Formulations were prepared using stoichiometric equimolar ratios of diclofenac sodium, Eudragit EPO and one of four fatty acids, namely lauric acid, myristic acid, palmitic acid and stearic acid. Mass of each ingredient is expressed in grams for the preparation of one batch of PEC microparticles.

Formulation	Ingredients	Fatty Acid Chain Length	Diclofenac Sodium (g)	Eudragit EPO (g)	Fatty Acid (g)
DS-EE-LA	Diclofenac–Eudragit EPO–Lauric acid	C12	0.3181	0.3181	0.2003
DS-EE-MA	Diclofenac–Eudragit EPO–Myristic acid	C14	0.3181	0.3181	0.2284
DS-EE-PA	Diclofenac–Eudragit EPO–Palmitic acid	C16	0.3181	0.3181	0.2564
DS-EE-SA	Diclofenac–Eudragit EPO–Stearic acid	C18	0.3181	0.3181	0.2845

**Table 2:** Accuracy and precision of the HPLC method for diclofenac sodium (DS). Percent recovery of DS at three concentration levels (25%, 50%, and 95% of the calibration range) is shown for three independent runs, with mean recovery values reported (*n* = 3). Predicted concentrations are presented as mean ± SD (*n* = 3) at five nominal concentration levels, with relative standard deviation (%RSD) values reported to assess intra-day and inter-day precisions.

Actual Concentration of DS (µg/mL)	Run 1	Run 2	Run 3	% Mean Recovery (*n* = 3)
	% Recovery	% Recovery	% Recovery	
12.5	103.26	103.47	103.26	103.33
25	101.36	101.47	101.36	101.40
47.5	98.93	98.30	98.59	98.60
**Actual Concentration (µg/mL)**	**Intra-Day**		**Inter-Day**	
	**Predicted Concentration (µg/mL, Mean ± SD, *n* = 3)**	**%RSD**	**Predicted Concentration (µg/mL, Mean ± SD, *n* = 3)**	**%RSD**
10	9.98 ± 0.02	0.16	9.85 ± 0.10	1.05
20	19.48 ± 0.04	0.21	19.30 ± 0.12	0.61
30	29.62 ± 0.06	0.19	29.03 ± 0.46	1.58
40	39.72 ± 0.04	0.11	39.14 ± 0.47	1.19
50	50.45 ± 0.06	0.11	49.26 ± 1.01	2.05

**Table 3:** DS content expressed as percentage of labelled drug content (mean ± SD, *n* = 3) for diclofenac sodium–Eudragit EPO–fatty acid (DS–EE–FA) complexes at baseline and after storage under ambient conditions, protected from light. Formulations were prepared with lauric acid (DS-EE-LA), myristic acid (DS-EE-MA), palmitic acid (DS-EE-PA), and stearic acid (DS-EE-SA). Baseline values were determined at 7 days post-manufacture when samples were first placed in capped glass vials for storage.

Polyelectrolyte Complex Formulation	Drug Content (% Labelled Content, *n* = 3, mean ± SD) After Storage at Ambient Conditions for Specified Periods		
	Baseline	1 Month	3 Months
DS-EE-LA	95.3 ± 0.3	99.0 ± 0.6	99.0 ± 0.5
DS-EE-MA	103.9 ± 1.5	102.0 ± 0.9	102.7 ± 0.2
DS-EE-PA	101.0 ± 0.2	95.7 ± 0.8	95.9 ± 0.4
DS-EE-SA	99.4 ± 0.3	97.5 ± 0.1	96.6 ± 0.2

**Table 4:** A comparison of the Medicine Acceptability Score (MAS) and binary response (Yes/No) for the samples evaluated by 25 participants in the taste evaluation study. Samples tested were diclofenac sodium (DS) and DS–Eudragit EPO–fatty acid (DS–EE–FA) complexes with lauric acid (DS–EE–LA), myristic acid (DS–EE–MA), palmitic acid (DS–EE–PA), and stearic acid (DS–EE–SA). Participants were categorised as promoters if they scored 8–10, passives if they scored 5–7, and detractors if they scored 0–4. MAS is calculated as (% promoters − % detractors).

(a) ‘Taste’ Data						
Sample	% Detractors	% Passive	% Promoters	MAS	% ‘Yes’	% ‘No’
DS	92	8	0	−92	20.8	79
DS-EE-LA	92	8	0	−92	29.2	71
DS-EE-MA	52	36	12	−40	60.0	40
DS-EE-PA	8	14	3	−5	92.0	8
DS-EE-SA	52	40	8	−44	72.0	28
**(b) ‘Willingness to Take Again’ Data**						
**Sample**	**% Detractors**	**% Passive**	**% Promoters**	**MAS**	**% ‘Yes’**	**% ‘No’**
DS	84	12	4	−80	20.8	79
DS-EE-LA	84	16	0	−84	29.2	71
DS-EE-MA	40	40	20	−20	60.0	40
DS-EE-PA	24	52	24	0	92.0	8
DS-EE-SA	32	52	16	−16	72.0	28

## Data Availability

The original contributions presented in the study are included in the article, further inquiries can be directed to the corresponding author due to ethical restrictions.
